# Unraveling the Role of Allo-Antibodies and Transplant Injury

**DOI:** 10.3389/fimmu.2016.00432

**Published:** 2016-10-21

**Authors:** Yoshiko Matsuda, Minnie M. Sarwal

**Affiliations:** ^1^Department of Surgery, University of California San Francisco, San Francisco, CA, USA

**Keywords:** HLA antibody, donor-specific HLA antibody, non-HLA antibody, antibody-mediated rejection, humoral immune system, *in vitro* B cell assay

## Abstract

Alloimmunity driving rejection in the context of solid organ transplantation can be grossly divided into mechanisms predominantly driven by either T cell-mediated rejection (TCMR) and antibody-mediated rejection (ABMR), though the co-existence of both types of rejections can be seen in a variable number of sampled grafts. Acute TCMR can generally be well controlled by the establishment of effective immunosuppression (1, 2). Acute ABMR is a low frequency finding in the current era of blood group and HLA donor/recipient matching and the avoidance of engraftment in the context of high-titer, preformed donor-specific antibodies. However, chronic ABMR remains a major complication resulting in the untimely loss of transplanted organs (3–10). The close relationship between donor-specific antibodies and ABMR has been revealed by the highly sensitive detection of human leukocyte antigen (HLA) antibodies (7, 11–15). Injury to transplanted organs by activation of humoral immune reaction in the context of HLA identical transplants and the absence of donor specific antibodies (17–24), strongly suggest the participation of non-HLA (nHLA) antibodies in ABMR (25). In this review, we discuss the genesis of ABMR in the context of HLA and nHLA antibodies and summarize strategies for ABMR management.

## Introduction

Organ transplantation improves the quality of life of patients with terminal dysfunction of organs, such as the kidney and pancreas, and is the most effective life support treatment for patients with heart, lung, and liver failure.

Although short-term prognoses for transplanted organs have improved significantly, long-term prognosis after 5–10 years remains insufficient, and reportedly reflects injury from chronic, indolent injury from sub-clinical antibody-mediated rejection (ABMR) (3–5, 15). Acute ABMR is a declining problem in organ transplantation as donor/recipient matching has improved (7, 16) and early acute ABMR is seen usually only in the context of ABO incompatible organ transplants (17, 18), and transplantation in highly sensitized patients with preformed donor-specific HLA antibodies (DSAs). Accordingly, preformed DSA are more likely to be produced before transplantation with histories of complications, such as pregnancy, previous transplant, blood transfusion, and prior organ transplantation (7, 19, 20). Hyper acute rejection, which can occur in the presence of preformed DSA, can be controlled using recently developed desensitization therapies (7).

Rejection due to *de novo* DSAs remains a major cause of transplanted organ loss, in the context of sub-clinical, chronic ABMR (21–24). Moreover, ABMR has also been reported in the absence of DSAs, leading to the discovery of specific non-HLA (nHLA) antigens that activate humoral immune responses in the graft. Potentially, nHLA antibody-mediated humoral immune responses develop acutely and chronically following transplantation and these antibodies may influence prognoses by participating in the onset and sequelae of rejection (16–18, 25–33). Although graft rejection has been reported among patients with nHLA antigens, one of challenges has been the discovery of the identity of these novel nHLA antigens and to correlate their presence and titers with ensuing mechanisms of transplant rejection.

## Molecular Pathophysiology

During ABMR, antibodies for donor antigens are produced following activation of humoral immune responses, involving activated T cells and complement pathways.

**Figure 1:**
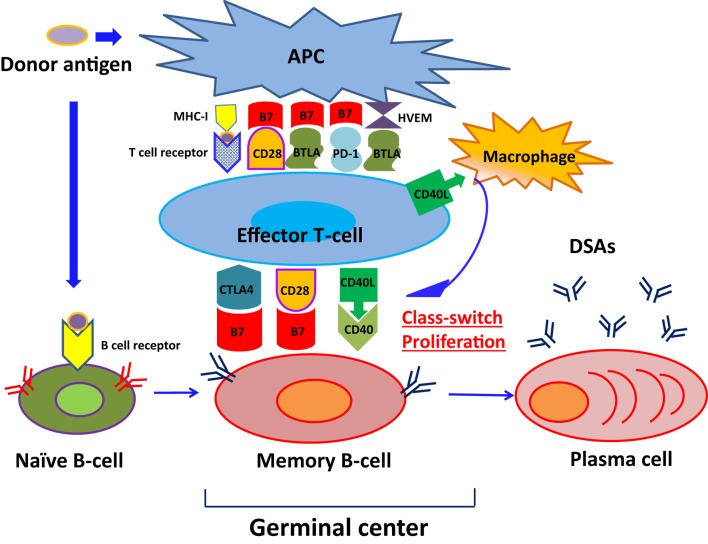
**The pathway of naïve B-cell differentiation into DSA-specific PCs**. Naïve B cells differentiate into DSA-specific plasma cells (PCs) via germinal centers following exposure to antigens, herpes virus entry mediator; HVEM.

As shown in Figure 1, naïve B cells differentiate into DSA-specific plasma cells (PCs) via germinal centers following exposure to antigens. This process involves initial uptake and surface presentation of donor antigens on antigen-presenting cells (APC) in response to an encounter of donor antigens, leading to activation of CD4^+^ effector T cells (34) and successive promotion of class-switching of naïve B cells and differentiation of memory B cells into PCs (35). Transmission of CD4^+^ effector T cell signals to B cells primarily involves association of major histocompatibility complex 1 (MHC-I) with T cell receptors (36). In addition, subordinate signaling pathways are activated by binding of CTLA4 (CD152), CD28, and CD40 ligand (CD40L) on T cell surfaces to the B7 (CD80/86) complex and CD40 on B-cell surfaces. Although CTLA-4 binds to B7, it reportedly downregulates T cell activity by binding to B7 with much greater affinity than CD28 (37–40). Intracellular CTLA-4 was closely related to the suppressor function of regulatory T cells (41–43) and reported the close relationship with autoimmune disease, including Graves’ disease, type 1 diabetes mellitus (DM) (44–48).

CD28 is expressed on CD4^+^ effector T cells and naive T cells (47), and promotes interleukin (IL)-2 production from B cells following binding to B7 complexes (48), leading to sustained naïve B cell differentiation into memory B cells (49). Conversely, CD40L mediates the class-switch of B cells in the germinal center by binding to CD40 expressing B cells (50) and support CD4^+^ effector T cells to help B cell differentiation (51, 52). Previous studies by Ettinger et al. (53) also showed that IL-21 induced PC phenotypes of human naïve and memory B cells following stimulation through B cell receptor (BCR) and CD40. Therefore, DSA-specific PCs developed and produced DSAs.

## The Role of Costimulatory Pathway in the Clinical Field

CTLA-4Ig (immunoglobulin) binds to B7 and then suppresses the engagement of CD28. CTLA-4Ig can suppress the function of activated T cells through regulatory T cells, which may help suppress established chronic inflammatory disease (54).

In the field of transplantation, Belatacept, which links to the extracellular domain of CTLA-4, has been approved for the treatment of acute kidney rejection. In addition, an important problem in the field is to control antigen-specific memory B cells differentiation into PCs. The infusion of Belatacept might suppress DSAs development in a T-cell-independent manner because it has been reported that the infusion of CTLA-4Ig 1 week or more after transplantation could prevent DSAs development in a fully mismatched mouse cardiac transplant model but did not affect T-cell function (55). In addition, in the recent clinical BENEFIT trial of Belatacept induction by Vincenti et al. (56), there was a significant lower incidence of DSA development with Belatacept induction, when compared to the standard CNI arm, despite the higher incidence of acute rejection seen early with Belatacept induction.

The results might indicate that CTLA-4Ig could inhibit the growth and survival of DSA-specific memory B cells or PCs in a human model. About the other suppressive receptors related to CD28, PD-1 (programed death-1) has been reported to be expressed on the surface of T cells, and B and T lymphocyte attenuator (BTLA) has been reported to be expressed on the surface of both B and T cells, both of which have also been attracting attention as targets for treating autoimmune diseases and cancer (57).

In the field of autoimmune disease, the involvement of signaling through CD40–CD40L interaction in autoimmune diseases has been reported and dysregulation of CD40 may induce macrophage-mediated coronary artery disease (CAD); the blockade of CD40L may, thus, be an attractive therapeutic target to improve CAD (58). Recent studies have also implicated altered regulation of the CD40 axis and generation of pathogenic activating anti-CD40 antibody for the generation of podocyte injury in focal segmental glomerulosclerosis (FSGS) recurrence after kidney transplantation (59, 60). Further research is needed to better elucidate how the CD40 axis may help control other autoimmune diseases.

## Human Leukocyte Antigen Antibodies

Histocompatibility analyses using cross-match, human leukocyte antigen (HLA) typing, and antibody tests are widely performed prior to transplantation in many laboratories, and are an accepted approach for limiting organ rejection. Recent developments in laboratory procedures, survey equipment, and technologies have led to highly sensitive detection of HLA antibodies.

Therefore, we could detect a very small amount of HLA antibodies and determine these antibody specificities; trace quantities of HLA antibodies recently provided useful prognostic information for ABMR and transplanted organ outcome and a judgment of transplant evaluation (61–65).

Major histocompatibility complex 1 class 1 (HLA-A, -B, and -C) and MHC class 2 (HLA-DR, -DP, and -DQ) have been identified as HLA antigens, and HLA antibodies can be detected in sera using FlowPRA^®^ Class I & II Screening Tests (One Lamda) to identify Class I or/and Class II HLA antibodies. In further analyses, positive cases should be identified using HLA LAB Screen HLA Class I or/and Class II single antigen beads (One Lambda) with Luminex technology, which determines antibody profiles against HLA Class I or Class II and indicates the presence or absence of DSAs.

## The Role of Preformed DSA in the Pathogenicity of Graft Injury

Donor-specific HLA antibodies that cause ABMR have been classified as those that are present before transplantation as well as those *de novo* that are produced after transplantation. Previous studies on kidney, heart, lung, and liver transplantation indicate that poor-prognosis is associated with the presence of DSAs before transplantation. We will next discuss the role of preformed DSAs in each organ transplant. With regard to kidney transplants, preformed DSAs have been recognized as one reason of hyper acute rejection. DSAs with high threshold MFI and DSAs with cross match-positive could predict ABMR onset after transplantation (7). With regard to pancreas transplants, we found a report describing that preformed DSAs did not affect graft prognosis (66) but DSAs could be detected from the sera with significantly higher probability than in recipients without a history of preformed DSAs after transplant. As a result, recipients sensitized by DSA before transplant had a history of DM more than 10 years after the transplant, so we should pay more attention to postoperative management, including blood sugar management (67).

With regard to liver, heart, and lung transplants, it is already known that preformed DSAs could affect graft outcome and patient mortality. In addition, preformed C1q binding DSAs have been reported to affect graft prognosis in liver and heart transplants and preformed DSAs with MFI ≥5000 and IgG3 DSAs could be risk factors for ABMR onset in liver transplant cases (19, 68, 69). Indeed, additional risk factors for ABMR in these patients include ABO incompatible and cross match-positive status, cases with a history of previous transplants, pregnancy, and blood transfusions (19, 70, 71). With regard to lung transplants, preformed DSAs have been reported to promote *de novo* DSA development early after transplant and patient survival (72–75). Therefore, these data on clinical correlations of DSA and rejection in different organ transplants suggests that improved screening and therapies, such as desensitization before transplant, may be of benefit across different types of solid organ transplants to limit subsequent postoperative complications (76).

### Mechanisms of Onset of ABMR by Preformed DSA

Antibody-mediated rejection caused by preformed DSAs manifests as hyper acute rejection immediately after transplantation, leading to failure of the transplanted organ within several hours. In these cases, DSAs immediately bind to all capillary endothelium surfaces in the transplanted organ, and concomitant complement activation leads to the formation of fibrin clots and acceleration of blood coagulation. Subsequently, rapid peripheral circulation incompetence causes necrosis of vascular walls, intense bleeding of the transplant, and necrosis in neighboring tissues. Finally, inflammatory cells, such as neutrophils, infiltrate capillary endothelial surfaces, and further undermine the transplant (6, 77).

### Management of ABMR by Preformed DSA

Improvements in desensitization therapy have enabled management of high risk recipients, such as those with cross match-positive phenotypes and high organ transplantation sensitivity; as a result, the prevalence of severe hyper acute rejection by preformed DSAs has decreased significantly (7). Accordingly, Ng et al. summarized desensitization protocols and complications using rituximab, bortezomib, eculizumab, and alemtuzumab, and reported promising graft survival in patients across various institutes (78). However, complications included anemia and thrombocytopenia, likely reflecting myelosuppression by these agents. In addition, various infections in some cases were detected, including cytomegalovirus (CMV), BK virus, and Epstein–Barr virus (EBV), indicating that desensitization therapy disposes patients to an increased risk of opportunistic viral infections. In addition, it was reported that induction with T-cell depleting agents (anti-thymocyte globulin) was closely associated with CMV, EBV, and BK polyomavirus (BKV) infections in comparison with IL-2a receptor antagonists (anti-CD25) (79). Therefore, the use of T-cell depleting agents should be avoided as an immunosuppressive reagent or induction. If possible, the use of IL-2a receptor antagonists or no induction should be considered (79, 80). Additionally, it was expected that these virus infection may contribute to the activation of immune responses in transplanted organs, and dose reductions of immunosuppressive agents may activate immune reactions to graft antigens.

To address this issue, prediction and early detection of viral infections is critical, and could be used to inform doses reductions of immunosuppressive agents. Concomitant administration of preventive and therapeutic antiviral agents is also critical in the management of these patients.

### Desensitization Therapy

Prior to the introduction of rituximab, plasmapheresis and splenectomy were long recommended as desensitization therapies for patients with ABO incompatible kidney transplants. Subsequently, rituximab was shown to inhibit the onset of ABMR without splenectomy. Rituximab is a monoclonal antibody (mAb) against the protein CD20, which is expressed in immature and mature B cells. However, because CD20 is not expressed on PCs, rituximab may not inhibit the production of DSAs by PCs. In addition, recent studies show varying effects of rituximab on B cell phenotypes, with higher sensitivity of naïve B cells than memory B cells (81). Thus, although rituximab suppresses immune activation and may not provide protection from infection, memory B cells may remain viable.

In addition, posterior reversible encephalopathy syndrome (PRES) and acute respiratory distress syndrome (ARDS) was reportedly increased in patients treated with rituximab as severe adversity effect (82). These data warrant further clarification of the depletion mechanisms of rituximab in B cells.

Bortezomib is a proteasome inhibitor that was developed as a treatment for multiple myeloma, and the effectiveness of this agent against transplant rejection was reported in 2008. These studies showed downregulated immune responses to donor antigens, recovery of graft function, and long-term suppression of serum antibody levels. However, inhibition of the proteasome by bortezomib may be detrimental to healthy cells (83–88).

As an alternative, eculizumab is a recombinant humanized monoclonal IgG2/4 antibody that suppresses complement activation and inhibits production of C5, which is the final product of the complement pathway and activates inflammatory responses and ultimately results in apoptosis of infected cells (89). Accordingly, treatments with this agent led to severe infectious diseases, including meningitis (90).

Finally, alemtuzumab is a recombinant DNA-derived humanized IgG1 kappa mAb that is directed toward CD52 and is used is used to treat B-cell chronic lymphocytic leukemia (B-CLL) and multiple sclerosis patients, warranting consideration for the treatment of ABMR. As adverse effect, it has been associated with infusion-related events (91, 92).

### Infection as a Trigger of Rejection

#### Cytomegalovirus Infection as a Trigger of Rejection

Cytomegalovirus is among the most common infections after solid-organ transplantation, and results in significant morbidity, graft loss, and adversity. Although numbers of CMV-seronegative (R^−^) cases have increased recently in healthy subjects, those with organ transplants from CMV-seropositive donors (D^+^) are at the highest risk of primary CMV disease, which can easily become serious causing the reactivation of latent virus transmitted in the allograft (93, 94). Additionally, a close relationship between CMV infection and allograft rejection has been reported in CMV D^+^/R^−^ liver and kidney transplant patients (93, 95).

##### Laboratory Diagnosis of CMV

###### Nucleic Acid Testing

Nucleic acid testing (NAT) is widely used to detect and quantify CMV RNA and DNA.

###### Serology

Serological analyzes allow risk stratification of patients during the pre-transplant screening phase on the basis of tests for CMV IgG antibodies in both donors and recipients, and can indicate the presence of latent infection.

###### Antigenemia

The antigenemia assay detects the CMV pp65 antigen in infected leukocytes from peripheral blood, and has been used for rapid diagnosis of CMV infections in transplant recipients (96).

##### Treatment of CMV

In a previous study, valganciclovir was found to be more effective than oral ganciclovir at preventing CMV disease in solid organ transplant recipients (97), suggesting that extension of valganciclovir prophylaxis to 200 days may benefit high risk (D^+^/R^−^) kidney recipients. Following transplantation, CMV disease is predominantly treated using intravenous (IV) ganciclovir (5 mg/kg every 12 h) and oral valganciclovir (900 mg twice daily) (98).

#### BK Polyomavirus Infection as a Trigger of Rejection

More than 90% of healthy subjects become infected with BKV (99, 100), which is the major cause of polyomavirus-associated nephropathy (Py-VAN) and presents a 1–15% risk of allograft failure in kidney transplant patients (101–106). And it has been reported that BKV-activated antibody reactivity in recipients at the onset of immunosuppression (107). However, although number of BKV-seronegative (R^−^) cases has increased recently in healthy subjects, these patients are the most susceptible to BKV disease following transplantation from BKV seropositive donors (D^+^) (108, 109).

##### Laboratory Diagnosis

Screening for BKV replication should be performed at least every 3 months during the first 2 years after transplantation, and then annually until the fifth year.

###### Nucleic Acid Testing

Nucleic acid testing in polymerase chain reaction (PCR) is used to detect amplifications of BK DNA.

###### Urine Cytology

Urine cytology is sufficient to detect decoy cells, which are associated with BKV induced organ failure.

##### Treatment of BKV

First, reduction of immunosuppression should be considered (110, 111). In patients with sustained high-level plasma BKV loads despite dose reductions of immunosuppression agents, administration of antiviral agents (*Cidofovir)*, and a replacement for mycophenolic acid (*Leflunomide)*, intravenous immunoglobulin (IVIG), and anti-mycotic agents (*Fluoroquinolones*) should be considered.

#### Epstein–Barr Virus Infection as a Trigger of Rejection

Epstein–Barr virus contributes to the pathogenesis of post-transplant lymphoproliferative disease (PTLD) occurring cases early after transplantation in more than 90% of the cases, and small intestine transplantations are associated with higher risks than heart, lung, and liver transplantations (112, 113). The close relationship between EBV infection and ABMR has been reported in heart transplantation (114).

##### Laboratory Diagnosis

###### Nucleic Acid Testing

Epstein–Barr virus DNA monitoring for EBV D^+^/R^−^ recipients should be recommended, with continued EBV load screening every 3–6 months until 2–3 years after transplantation. This monitoring is particularly important for EBV-seropositive recipients with intestinal transplants, and monitoring of EBV DNA every 2–4 weeks in the first 3 months should be performed, monthly until 6 months post-transplantation, and then every 3 months until the end of the first year.

##### Treatment of EBV

Antiviral prophylaxis for high risk patients (EBV D^+^/R^−^) is considered in some centers (99).

Treatment with acyclovir, ganciclovir, and IVIGs has shown some benefits in the prevention of PTLD among EBV-seronegative recipients who their donors are EBV-seropositive (113).

## The Role of *De Novo* DSA in the Pathogenicity of Graft Injury

**Figure 2:**
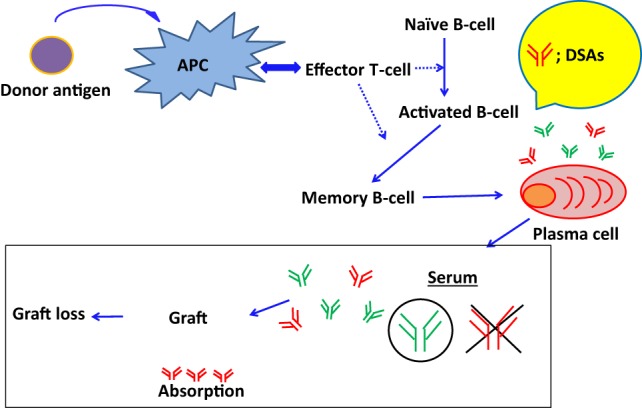
**The absorption of DSAs into graft and the development of ABMR**. DSAs may be absorbed into transplanted organs during the early phases of antibody production.

Recent reports show that DSAs play an important role in ABMR onset, and this has been shown by highly sensitive monitoring of HLA antibodies in the sera (11–13). However, DSAs may be absorbed into transplanted organs during the early phases of antibody production (115) (Figure 2). Accordingly, in the Guidelines of the Transplantation Society (TTS), post-kidney transplant DSA monitoring is not recommended for all patients beyond the first year (116). Hence, to avoid the influence of absorption, antibody production from PCs has been analyzed *in vitro*, because these antibodies may not be influenced by the absorption and provide us with further detailed illustrations that are available to clarify how these antibodies are produced in organ recipients. However, PCs are seldom found in blood and predominate in bone marrow and secondary lymphoid tissues, techniques for differentiating B cells into PCs, are required to investigate antibody production (117–119).

### *In Vitro* B Cell Assays

Determination of HLA antibodies in supernatants of cultured B cells can better inform ABMR management than those in sera. Therefore, some researchers have suggested that if peripheral B cells could be differentiated into PCs *in vitro*, then the *in vitro* differentiation of peripheral B cells into PCs may facilitate the control of ABMR. However, unlike *in vitro* T cell assays that have long been used to control T cell-mediated rejection (TCMR), primary cultures of B cells are difficult to maintain and *in vitro* B cells assays have not long been established. Although memory B cells were reportedly differentiated into APC *in vitro* (120), these short comings require further improvements in the ease and convenience of B cell culture, and subsequent assay development that can be used to detect HLA antibodies in B cell culture supernatants to ultimately prevent transplant rejection.

Moreover, there are important points to detect HLA antibodies from the B cell culture supernatant. Peripheral B cells include naïve B cells and memory B cells (121, 122); and these B cells derived from PCs survive for varying durations and produce antibodies. However, HLA antibodies that cause ABMR are mainly produced by memory B cells (123–125), warranting establishment of *in vitro* B cell assays in which memory B cells are selectively differentiated into PCs *in vitro* and are used to produce antibodies. In addition, many reports have showed that long-lived PCs, which produce antibodies in the bone marrow for long periods, play an important role in ABMR (126–129). Therefore, to monitor the progression of antibody-mediated diseases, *in vitro* culture systems, in which B cells are differentiated to their terminal stage (long-lived PCs), are urgently required. On using a clinical specimen, the volume of B cells in peripheral blood is very low following immunosuppression or particularly when we could collect B cells from recipients who experienced desensitization therapy.

However, feeder cells can strongly activate human B cells to proliferate and differentiate in a cell–cell contact-dependent manner in these cases. Thus, *in vitro* B cell assays of HLA antibodies from the cultured supernatants may lead to drug sensitivity tests that are similar to those for T cells, may contribute to clinical applications of personalized immunosuppression, and the development of new immunosuppressant agents that control ABMR.

### *In Vitro* Memory B Cell Assays

We found a report about immunosuppressive agent susceptibility for the differentiation of human B (CD19^+^) cells *in vitro* with a combination of IL-21, phosphorothioate CpG-oligodeoxynucleotide (CpG-ODN), histidine-tagged soluble recombinant human CD40 L and anti-polyhistidine mAb (130). IL-21 is produced by follicular helper T cells (131), which synergistically induce maximum Blimp-1 upregulation and optimal PC differentiation with CD40 L (132). TLR9 agonist CpG-ODN activates B cells proliferation and promotes PCs differentiation (133). This culture system induced IgG production but could not sustain the survival of PCs for a long period. It might indicate that the other cytokines play an important role in human B cells differentiation into mature PCs *in vitro*, because other groups have reported that APRIL and the B cell activating factor (BAFF) would support the survival of PBs and PCs recently (134–137). In addition, a previous report has shown that CD27^+^ memory B cells could be differentiated into long-lived PCs with supernatants from bone marrow stromal cell line M2-10B4 (138), which support the long-term culture of human bone marrow stem cells. The mechanisms by which M2-10B4 cells contribute to PCs survival has yet to be revealed, but it is suggested that CD27^+^ memory B cells demand well-balanced support from stromal cells (139–142). In addition, different environments or signal transmission might be required for the differentiation of CD27^−^ naïve and CD27^+^ memory B cells into mature PCs. Therefore, we should improve the *in vitro* B cell assay to sustain CD27^+^ memory B cell-derived PC survival for a long-term selectively. For example, we should examine how any humoral factors, including growth factor or any cytokines from activated T cells could affect CD27^+^ memory B cell growth and survival *in vitro*, while referring to the reports that helper T-cells may mediate CD27^+^ memory B cell differentiation into PCs *in vivo* (143).

### Risk Factors of ABMR from *De Novo* DSA

Not all DSAs participate in ABMR and transplanted organ prognosis (7), and although C1q binding DSAs are reported risk factors for ABMR onset, further studies of DSA characteristics are required to identify those with prognostic value. In addition, various other factors influence transplanted organ prognoses (ABMR onset, graft survival) and require further investigation. About the risk factors for graft loss, thrombotic microangiopathy (TMA), glomerulopathy, C4d deposition, and chronic injury change in histopathological diagnoses were reported.

As other factors besides histopathological findings, a history of subclinical ABMR and TCMR and a decline of graft function could be risk factors. This might indicate that a graft would fail with high probability when the humoral immune response toward a donor-specific antigen has proceeded to an irreversible stage.

#### C1q Binding DSA

C1q appear in the beginning of the classical complement pathway, and C1q binds directly to antigens and initiates classical complement pathway activation. Subsequent C1q-activated reactions include (i) antigen binding, (ii) binding to C-reactive protein, and (iii) binding to antigen–antibody complexes, and can lead to the activation of C3 convertase and the degradation of C3 to C3b and C3a (144, 145). Of these, C3b is the main effector of the complement pathway, while C3a activates inflammatory responses. Indeed, C1q may play important roles in the activation of inflammatory reactions against grafts. Accordingly, C1q binding to DSAs reportedly influences the frequency of ABMR onset and glomerulopathy in solid organ transplants, leading to increased chances of graft failure. Thus, binding of C1q to DSAs may be highly predictive of graft prognosis, warranting the development of interventions that decrease the presence of C1q binding DSAs. The C1qScreen™ (One Lamda) is a reliable tool for distinguishing complement-binding antibodies from non-complement-binding ones, and is widely applied using Luminex-based LABScan™ 100 flow fluorescence analyzers to determine relative amounts of C1q binding antibodies. The C1qScreen™ in combination with the Luminex-based LABScan™ can indicate the relative amount of C1q bound to DSAs and provides us with useful information from the sera (146). In addition, in C1q-positive cases despite being DSA-negative, graft survival is poor. It suggested that C1q could affect the transplanted organ prognosis by itself.

#### DSA Characteristics

In this study, we tabulated previously reported factors that participate in ABMR and graft loss (Table 1). Reports show that DSAs with higher mean fluorescent intensities (MFI) of ≥15,000 cause ABMR with higher probability than those with MFI of ≤5000, and higher level of DSAs may activate humoral immune reactions to donor antigens. In addition, many papers indicated that class II DSAs should be considered as a risk factor, particularly at the onset of ABMR. However, DSA specificities that activate humoral immune response to donor antigen may depend on the type of transplanted organ, and the further recognition about detailed association between DSAs and graft outcome is required in each solid organ transplantation.

**Table 1:** **Various other factors influence transplanted organ prognoses and require further investigation**.

		Risk factors	Out come	
			
Study size	Organ	ABMR	Graft loss	Reference
226	Kidney	Highly sensitized patients	ABMR-positive	(147)
		DSA relative intensity scores greater than 17	Thrombotic microangiopathy (TMA) positive	
		Presence of both class I and II DSAs at transplant	Induction with intravenous immunoglobulin and rituximab	
62		C1q-positive	C1q-positive	(148)
			Both of DSA- and C1q-positive	
			Transplant glomerulopathy	
			Decline of eGFR	
1016		Complement-binding DSA DSA-positive	Complement-binding DSA	(149)
			DSA-positive	
1307			Subclinical ABMR	(150)
			Subclinical TCMR	
1365		TCMR	TCMR diagnosed after the first year post-transplant	(151)
			Chronic histological injury	
			Transplant glomerulopathy	
67 (grafts)			Late aABMR	(152)
885			Capillary C4d-positive	(153)
1054		TCMR	Higher glomerulitis scores	(154)
			Higher C4d staining scores	
1			Plasma cell-rich rejection (PCRR) with ABMR	(155)
237		DSA-positive preformed DSA-positive	DSA-positive	(7)
			AMR	
			DSA-positive/CXM-positive	
234			Microcirculation inflammation	(4)
274			C1q-fixing DSAs	(140)
152	Pancreas-kidney		*De novo* DSA-positive	(67)
439	Pancreas		Elevated DSA	(156)
			Preformed DSA-positive	
2631	Liver	Preformed class II DSAs positive MFI ≧5000		(19)
1270		Preformed C1q-fixing class II DSA	IgG3 DSA-positive	(157)
			*De novo* IgG3 DSA	
749			*De novo* DSA development	(158)
15	Heart	SAB-C1q-positive DSA CDC-XM-positive		(9)
243			*De novo* DSA-positive	(159)
			Persistent DSA	(160)
44	Lung	DSA-positive	HLA-DQ DSA (>10,000)	(71)
60				
546			Early anti-HLA class II DSA	(72)
			Pre-operative HLA antibodies	
			Retransplantation	
			Postoperative PGD	
79	Intestine		*De novo* DSA development early after transplant	(161)
291		DSA-positive	DSA-positive	(162)

With regard to kidney transplants, DSAs with high threshold MFI and C1q binding DSAs have been reported to be closely related to TMA, glomerulopathy, microangiopathy, C4d deposition, extensive interstitial fibrosis, and tubular atrophy and these factors could affect graft prognosis in the long term (147–154). With regard to pancreatic transplants, elevated DSAs could affect graft prognosis (67, 156). With regard to liver transplants, IgG3 DSAs and C1qbinding DSAs were related to graft survival and class II DSAs were shown to be closely related to acute rejection early after a transplant (157). In addition, DSAs could affect graft outcome and reduce graft survival 1 year or more after a transplant by itself (158). With regard to cardiac transplants, C1q binding DSAs and cross match positivity could be risk factors for ABMR and *de novo* DSA development and persistent DSA were found to be closely related to graft loss (9, 159, 160). With regard to lung transplants, DSA has been related to ABMR, cellular rejection, and bronchiolitis obliterans and could significantly reduce postoperative survival 3 years later compared with that in DSA-negative recipients. In addition, *de novo* DSA (along with HLA-DR mismatch) development has been reported to reduce postoperative survival (71, 159). With regard to intestine transplants, *de novo* DSA development early after transplant could affect graft prognosis and might be effective for screening of acute rejection because DSA measurement has been shown to be closely related to histological findings (161, 162). The characteristics of DSA that could affect graft prognosis vary among the different types of organ transplant; we should, thus, understand these features well and make use of them for the postoperative management of transplant recipients.

### Onset Mechanisms of ABMR from *De Novo* DSA

Antibody-mediated rejection caused by *de novo* DSAs typically appears several weeks to months after transplantation, but can develop at any time as far as a graft engrafts afterward.

Following the absorption of HLA antibodies onto capillary endothelial donor antigens (mainly HLA antigen), activation of pro-complement solidification and accumulation of inflammatory cytokines, macrophages, and neutrophils are caused successively. Therefore, it leads to microangiopathy and gradual annual declines in graft function (52, 163–165). Under these conditions, ABMR-mediated microangiopathy is chronic and sustained, although moderate inflammatory activities result in slow and irreversible disease progression.

### Management Strategies of ABMR by *De Novo* DSA

In a previous section, we suggested that immunosuppressive therapy limits differentiation of naïve B cells to germinal center B cells by controlling CD4^+^ effector T cell stimulation. However, stronger immunosuppressive therapy is required to control B cell growth and survival after differentiation of naive B cells to memory B cells in germinal centers. As a result, chronic use of immunosuppressive agents after differentiation of naïve B cells into memory B cells corresponding to HLA antibodies may no longer affect B cells in germinal centers.

**Figure 3:**
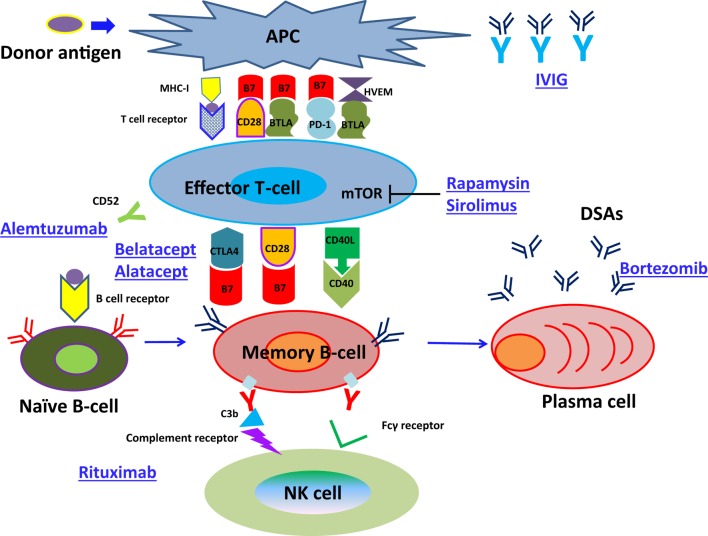
**The pathway of naïve B-cell differentiation into DSAs specific PCs and how immunosuppressive reagents suppress the development of ABMR**.

Thus, further attention should be paid to pancytopenia, anemia, and viral infection as well as to those concerning B cell differentiation, because more strong immunosuppressive therapy might be necessary to inhibit memory B cell growth and survival in comparison with naïve B cells. In particular, rituximab administration induces CD20^+^ memory B cell apoptosis (166), bortezomib therapy inhibits the production of DSAs production from PCs (167), and IVIG can be used to reduce circulating DSAs (168) (Figure 3). Hence, the development of new immunosuppressive agents that inhibit memory B cell growth and survival is warranted.

In addition, the development of a diagnostic method for predicting the development of DSA specific memory B cells as soon as possible has been required.

Therefore, the control of ABMR is very difficult; the disease state progresses irreversibly and severely and is unresponsive to increasing immunosuppression following diagnosis using currently available methods. Although graft tissue biopsies are the most reliable diagnostic method for ABMR, it was hard to perform frequently because it is very invasive. Therefore, less invasive diagnostic approaches are urgently required to predict the development of DSA-specific memory B cells.

Histological studies of ABMR following solid organ transplantation show that the classical complement pathway is activated after adhesion of DSAs to capillary endothelia, and that C4d produced and deposited as the final product of this pathway is an important ABMR diagnostic factor before (169). In late years, the number of reported cases of C4d-negative ABMR has recently increased (15) and some DSA-related mechanisms that are independent of the classical complement pathway have been identified. In each organ transplantation, these observations necessitate revision of histologic diagnostic criteria for all organ transplant patients, and improved the understanding of ABMR (170–172).

### Banff Score for Diagnosis of ABMR

Pathological diagnoses play important clinical roles, and diagnostic criteria have been revised for all organ transplants. In particular, Banff score are widely used as histologic methods for kidney transplantations, although diagnostic criteria were substantively revised in 2013; indeed, the roles of ABMR, DSAs, and C4d deposition in grafts received greater emphasis in the previous diagnostic criteria before the meeting in 2013. In the 2013 revised edition (Table 2), C4d-negative ABMR became the diagnostic focus especially, reflecting on the increased numbers of reported cases. Thus, we listed the important diagnostic criteria for ABMR in the revised edition in 2013, including confirmation of microangiopathy, evidence for DSA-capillary endothelial reactions, and detection of DSA in the serum. In particular, diagnosis of DSA-capillary endothelial reactions requires at least one of the following observations; (i) C4d deposition in peritubular capillaries (PTC), (ii) evidence of more than moderate microangiopathy and microvascular inflammation (MVI; g + ptc ≥ 2), and (iii) expression of endothelial activation (ENDAT) and injury transcripts.

**Table 2:** **Revised classification of antibody-mediated rejection**.

Acute/active ABMR	
1	Evidence of acute tissue injury, including one or more of the following
	Microvascular inflammation (g > 0 and/or ptc > 0)
	Intimal or transmural arteritis (v > 0)
	Acute thrombotic microangiopathy, in the absence of any other cause
	Acute tubular injury, in the absence of any other cause
2	Evidence of current/recent antibody interaction with vascular endothelium, including at least one of the following:
	**Linear C4d staining in ptc**
	**Moderate microvascular inflammation(g + ptc ≧ 2)**
	**Increased expression of gene transcripts indicative of endothelial injury**
3	Serologic evidence of DSAs
Chronic/active ABMR	
1	Evidence of chronic tissue injury, including one or more of the following
	Transplant glomerulopathy(cg > 0)
	Severe ptc basement membrane multilayering (requires EM)
	Arterial intimal fibrosis of new onset, excluding other causes
2	Evidence of current/recent antibody interaction with vascular endothelium, including at least one of the following
	**Linear C4d staining in ptc**
	**moderate microvascular inflammation(g + ptc ≧ 2)**
	**Increased expression of gene transcripts indicative of endothelial injury**
3	Serologic evidence of DSAs

*The bold font showed the most important factor to diagnose ABMR (Acute and Chronic)*.

*DSAs, donor-specific HLA antibodies; EM, electron microscopy*.

*Furthermore, in the revised criteria, ABMR phenotypes have been classified as acute/active; chronic/active corresponding to the diagnostic criteria, which have been listed in detail*.

Microvascular inflammation scores and C4d deposition in PTC are currently the most commonly used diagnostic criteria. However, according to this standard, ABMR diagnoses are recommended in the presence of strong MVI, even in specimens that are C4d-negative.

Thus, C4d deposition has not been necessary for ABMR diagnoses after the meeting in 2013.

**Figure 4:**
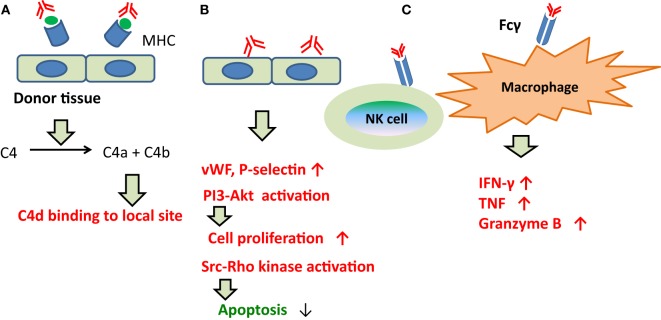
**The development of ABMR caused by DSAs**. **(A)** Indirect injury via complement fixation or recruitment. C1q was the assumed trigger of the classical complement pathway following binding of DSAs to capillary endothelia. Although C4d is the final product. **(B)** Direct injury to the capillary endothelium. DSAs may directly promote vascular endothelial cell growth and proliferation, and inhibit apoptosis in capillary endothelia. **(C)** Recruitment of inflammatory cells with Fc receptors. DSAs have been shown to bind with Fcγ on the cell membrane surfaces of macrophages, natural killer cells, and neutrophils, and to induce inflammatory cytokine production and microangiopathy.

By contrast, prior to 2013, ABMR was considered reflective of injury to capillary endothelia (165, 173), and C1q was the assumed trigger of the classical complement pathway following binding of DSAs to capillary endothelia. Although C4d is the final product (Figure 4), this classical pathway was not necessarily activated during ABMR in C4d-negative patients.

About the other pathways, DSAs may directly promote vascular endothelial cell (EC) growth and proliferation, and inhibit apoptosis in capillary endothelia (Figure 4); DSAs have been shown to bind with Fcγ on the cell membrane surfaces of macrophages, natural killer cells, and neutrophils, and to induce inflammatory cytokine production and microangiopathy (Figure 4).

Furthermore, in the revised criteria, ABMR phenotypes have been classified as acute/active, chronic/active corresponding to the diagnostic criteria, which have been listed in detail.

Among these, evidence of acute tissue injury as the diagnostic criteria of acute/active ABMR, and morphologic evidence of chronic tissue injury as the diagnostic criteria of chronic/active ABMR, is considered central. Therefore, effective management of ABMR entails varied treatments for differing levels of pathological progress, and these diagnostic criteria identify ABMR phenotypes with sufficient accuracy to inform treatments.

## The Role of Non-HLA in the Pathogenicity of Graft Injury

Rejection by nHLAs was previously recognized as an unexpected hyper acute rejection of HLA identical transplants (174–177). Recently, it has been accepted that nHLA antibodies play an important role in acute and chronic rejection (178–184).

Moreover, in a report from 1997, antibodies against nHLA antigens were shown to activate humoral immune responses to graft antigens and cause graft injury (185). Subsequently, graft loss due to immunological factors occurred in 56% of cases, and 38% of factors were nHLA. Thus, because the probability of graft loss due to nHLA factors was shown to be greater than that due to HLA factors (186), in late years, more attention has been paid to nHLA factors in the transplantation field. For instance, the former have received increased attention in the transplantation field with the anti-MHC class I chain-related gene A (MICA) antibodies and nHLA antibodies to ECs in the presence of complement, as identified in numerous recent reports. Thus, it was expected that humoral response toward nHLA antigens is primarily activated to donor antigen on ECs.

However, while MICA and ECs was not expressed on lymphocyte membranes and was undetectable using cross-match studies (10), the HLA antibody tests LAB Screen Mixed Class I & II and LAB Screen MICA Single Antigen have been successfully used to detect anti-MICA antibodies in sera.

In addition, there are many reports on the other nHLAs that were associated with ABMR.

These reports showed that the type of nHLA antigens differed between patients with hyper acute rejection, acute rejection, and rejection due to chronic allograft injury (CAI), and it may predict graft success and that management plans could be informed by mapping nHLA antigens in recipient sera before transplantation, further indicating the utility of serum nHLA determinations in the diagnosis and management of ABMR.

### Management Strategies of ABMR by Non-HLA

Previous studies have reported the detection of nHLA antibodies using ELISA and FACS. However, the clinical utility of these assays remains unclear, because they fail to distinguish antibodies from autoantibodies. Additionally, nHLA antibodies may be detectable in sera from patients with failed grafts but no immunological factors (26, 187). Thus, specific detection of nHLA antibodies that activate humoral immune responses to grafts requires more sensitive methods. For example, by using high-density protein array platforms (26), serum nHLA antibodies in transplant recipients may have up to 9000 different target proteins/antigens and these antibodies were screened immediately, indicating the importance of high throughput screening. In addition, nHLA genotyping of donor and recipient to estimate the risk of ABMR in recipients, such as HLA may be required; the specificities of these antibodies to nHLA should be identified in more details. For example, we should examine if nHLA has the capacity to bound complement or not and if these antibodies could activate humoral immune response toward the donor antigen by using donor-specific ECs.

Mechanism of participation of nHLA antibodies in ABMR and graft loss has not been investigated sufficiently. However, we could find reports that C4d deposition is related to ABMR causing from nHLA antibodies with high probability, indicating that this type of ABMR was caused by the activation of the complement classical pathway. Moreover, further studies are warranted to establish effective immunosuppressive therapies thereby clarifying the mechanism.

In addition, we summarized the association between the representative nHLA antibodies and graft prognosis (Table 3).

**Table 3:** **A list of selected nHLA antibodies and gene in transplantation**.

nHLA antibody (nHLA-ab)	Organ	Associated factors	Reference
Anti-protein kinase C zeta (PKCf) ab	Kidney	Graft loss	(188)
		Steroid-resistant rejection and the hypertension	
		Mononuclear cell infiltrate of acute rejection	
Anti-MHC I-related chain A (MICA) ab	Kidney	Poor graft survival with only MICA and significantly poor with both antibodies(MICA^+^/HLA^+^)	(189–197)
	Kidney	Preformed MICA antibodies contributes to increasing frequency of graft loss	
	Kidney	Chronic rejection, poor graft survival	
	Kidney	Graft rejection, poor 1-year graft survival	
	Kidney	Poor graft survival	
	Heart	The incidence of transplant coronary artery disease	
	Heart	No negative effect on graft survival	
	Liver	Late graft rejection	
Anti-angiotensin II type I receptor (AT1R) ab	Kidney	Refractory vascular rejection	(198–201)
	Kidney	Cronic kidney disease	
	Kidney	Graft injury, graft loss	
	heart	Cellular and Ab-mediated rejection and early onset of microvasculopathy	
Anti-endothelial antibodies (AECA)	Kidney	Cellular rejection	(177, 202–206)
	Kidney	Hyperacute rejection	
	Kidney	Graft rejection	
	Kidney	Acute rejection	
	Kidney	Microvascular damage	
	Heart	Early acute rejection	
Anti-endothelial-1 type A receptor (ETAR) ab	Kidney	Hyperacute rejection	(199, 207)
	Kidney	Poor graft function early after transplant, hyperacute rejection	
	Kidney	Graft injury, graft loss	
	Heart	Cellular and Ab-mediated rejection and early onset of microvasculopathy	
Anti-peroxisomal-trans-2-enoyl-coA-reductase (PECR) ab	Kidney	Transplant glomerulopathy	(79, 208)
Anti-PRKRIP1ab	Kidney	Cronic kidney disease	(32, 208)
Antivimentin ab	Heart	It did not correlate with early post-transplant rejection or graft survival	(32)
Non-HLA pigmy ab	Heart	Mortality	(209)
Antibodies against	Kidney	Acute ABMR	(210)
Endoglin			
Epidermal growth factor (EGF)-like repeats			
Discoidin I-like domains 3			
Intercellular adhesion molecule 4			
FMS-like tyrosine kinase-3 ligand			
Antibodies against	Kidney	Chronic allograft injury (CAI)	(26)
MIG			
ITAC			
IFN-c			
Glial-derived neurotrophic factor (GDNF)			
Collagen type V, K-α1-tubulin	Lung	Graft disfunction, bronchiolitis obliterans syndrome	(207)
**nHLA gene**			
FN-γ, IL-1B, IL-1RN, IL-2, IL-6, IL-7, IL-17, CCR9, ESR1, FAS Stem cell		GVHD ↑	(206)
IL-10, NOD2, toll-like receptors		GVHD ↑ or GVHD ↓	
VDR		GVHD ↑, mortality ↑	
CTLA4		Acute GVHD ↑, survival ↑	
IL-7R, CXCL10		Transplant-related mortality ↑	
IL-18		Transplant-related mortality ↓	
Il-23R		Acute GVHD ↓	
HLA-E		Chronic GVHD ↓	
IL-1A		Chronic and acute GVHD ↑, transplant-related mortality ↑	
CCl-2		Over roll survival ↓, transplant-related mortality ↑	
CXCL12		Hematological recovery ↑	
TGFβ		Acute GVHD ↓, over roll survival ↓	
HMGB1		Relapse ↑, relapse-related mortality ↑, transplant-related mortality ↑, over roll survival ↑, acute GVHD ↓, chronic GVHD ↑	
MICA		GVHD ↑	

## Conclusion

Absorption of DSAs has been regarded as the main cause of ABMR. However, numerous recent studies have characterized the involvement of nHLA antibodies, and have shown that DSA- and nHLA-mediated ABMR phenotypes likely require different management strategies.

Specifically, hyper acute rejections due to preformed DSAs may be avoided with improved desensitization therapy, while *de novo* DSA-mediated ABMR remains difficult to diagnose without invasive graft tissue biopsies prior to critical disease progression. Although the roles of nHLA antibodies have been identified in ABMR, ensuing that mechanisms remain insufficiently understood to inform improvements in management strategies. Moreover, because physiological nHLA antibodies are indistinguishable from those that are closely related to humoral immune reactions against graft antigens, highly sensitive methods that distinguish ABMR-relevant nHLAs are required for clinical diagnoses and management planning for organ transplant patients.

## Author Contributions

YM and MS designed and wrote the paper. MS provided excellent advices.

## Conflict of Interest Statement

The authors declare that the research was conducted in the absence of any commercial or financial relationships that could be construed as a potential conflict of interest.
